# Supplementary material for: How many people need to classify the same image? A method for optimizing volunteer contributions in binary geographical classifications
Source: PLoS One. 2022 May 19;17(5):e0267114. doi: 10.1371/journal.pone.0267114 (PMC9119495; doi:10.1371/journal.pone.0267114)
Supplement: Attachment — Submitted filename: Responses to reviews.docx [file pone.0267114.s002.docx]

| Reviewer | Comment | Response |
| --- | --- | --- |
| Editors | 1. Please ensure that your manuscript meets PLOS ONE's style requirements, including those for file naming. The PLOS ONE style templates can be found at  https://journals.plos.org/plosone/s/file?id=wjVg/PLOSOne_formatting_sample_main_body.pdf and  https://journals.plos.org/plosone/s/file?id=ba62/PLOSOne_formatting_sample_title_authors_affiliations.pdf | Thank you for pointing us toward these guidelines. We have tried to follow them as best possible. However, there are a few seeming contradictions with the online guidelines at https://journals.plos.org/plosone/s/submission-guidelines  For instance, it isn't fully clear if the short title should be included on the title page. We have left it there as we are not sure where else it should go.  Also, note that the Institute for Globally Distributed Open Research is not locality based, so there is no city, country, etc. for it. |
| Editors | 2. Please include the data sources used in the Data availability statement and Methods section. | We have modified these sections to include the DOI for the online repository where our dataset is now stored. |
| Editors | 3. In your Data Availability statement, you have not specified where the minimal data set underlying the results described in your manuscript can be found. PLOS defines a study's minimal data set as the underlying data used to reach the conclusions drawn in the manuscript and any additional data required to replicate the reported study findings in their entirety. All PLOS journals require that the minimal data set be made fully available. For more information about our data policy, please see <http://journals.plos.org/plosone/s/data-availability>.  Upon re-submitting your revised manuscript, please upload your study’s minimal underlying data set as either Supporting Information files or to a stable, public repository and include the relevant URLs, DOIs, or accession numbers within your revised cover letter. For a list of acceptable repositories, please see http://journals.plos.org/plosone/s/data-availability#loc-recommended-repositories. Any potentially identifying patient information must be fully anonymized.  Important: If there are ethical or legal restrictions to sharing your data publicly, please explain these restrictions in detail. Please see our guidelines for more information on what we consider unacceptable restrictions to publicly sharing data: http://journals.plos.org/plosone/s/data-availability#loc-unacceptable-data-access-restrictions. Note that it is not acceptable for the authors to be the sole named individuals responsible for ensuring data access.  We will update your Data Availability statement to reflect the information you provide in your cover letter. | We mistakenly believed we had uploaded this as a supplemental file, a process that failed due to the large size of the dataset. The dataset is now available on Zenodo, and the DOI is now given in the methods section and the data availability statement. |
| Editors | 4. Thank you for stating the following financial disclosure:  "This research was supported by an International Institute for Applied Systems Analysis postdoctoral fellowship to CFS and the project ‘Using Crowdsourcing and Gaming Approaches for EO4SD Services (GAME.EO)’ funded by the European Space Agency (contract no. 4000125186/18/1-NB) to SF. The funders had no role in study design, data collection and analysis, decision to publish, or preparation of the manuscript."  We note that one or more of the authors is affiliated with the funding organization, indicating the funder may have had some role in the design, data collection, analysis or preparation of your manuscript for publication; in other words, the funder played an indirect role through the participation of the co-authors. If the funding organization did not play a role in the study design, data collection and analysis, decision to publish, or preparation of the manuscript and only provided financial support in the form of authors' salaries and/or research materials, please do the following:  a. Review your statements relating to the author contributions, and ensure you have specifically and accurately indicated the role(s) that these authors had in your study. These amendments should be made in the online form.  b. Confirm in your cover letter that you agree with the following statement, and we will change the online submission form on your behalf:  “The funder provided support in the form of salaries for authors [insert relevant initials], but did not have any additional role in the study design, data collection and analysis, decision to publish, or preparation of the manuscript. The specific roles of these authors are articulated in the ‘author contributions’ section. | We have added the requested text to the cover letter. |
| Editors | 5. We note that Figure 2 in your submission contain satellite images which may be copyrighted. All PLOS content is published under the Creative Commons Attribution License (CC BY 4.0), which means that the manuscript, images, and Supporting Information files will be freely available online, and any third party is permitted to access, download, copy, distribute, and use these materials in any way, even commercially, with proper attribution. For these reasons, we cannot publish previously copyrighted maps or satellite images created using proprietary data, such as Google software (Google Maps, Street View, and Earth). For more information, see our copyright guidelines: http://journals.plos.org/plosone/s/licenses-and-copyright.  We require you to either (1) present written permission from the copyright holder to publish these figures specifically under the CC BY 4.0 license, or (2) remove the figures from your submission:  a. You may seek permission from the original copyright holder of Figure 2 to publish the content specifically under the CC BY 4.0 license.  We recommend that you contact the original copyright holder with the Content Permission Form (http://journals.plos.org/plosone/s/file?id=7c09/content-permission-form.pdf) and the following text: “I request permission for the open-access journal PLOS ONE to publish XXX under the Creative Commons Attribution License (CCAL) CC BY 4.0 (http://creativecommons.org/licenses/by/4.0/). Please be aware that this license allows unrestricted use and distribution, even commercially, by third parties. Please reply and provide explicit written permission to publish XXX under a CC BY license and complete the attached form.”  Please upload the completed Content Permission Form or other proof of granted permissions as an "Other" file with your submission.  In the figure caption of the copyrighted figure, please include the following text: “Reprinted from [ref] under a CC BY license, with permission from [name of publisher], original copyright [original copyright year].”  b. If you are unable to obtain permission from the original copyright holder to publish these figures under the CC BY 4.0 license or if the copyright holder’s requirements are incompatible with the CC BY 4.0 license, please either i) remove the figure or ii) supply a replacement figure that complies with the CC BY 4.0 license. Please check copyright information on all replacement figures and update the figure caption with source information. If applicable, please specify in the figure caption text when a figure is similar but not identical to the original image and is therefore for illustrative purposes only. The following resources for replacing copyrighted map figures may be helpful:  USGS National Map Viewer (public domain): http://viewer.nationalmap.gov/viewer/ The Gateway to Astronaut Photography of Earth (public domain): http://eol.jsc.nasa.gov/sseop/clickmap/ Maps at the CIA (public domain): https://www.cia.gov/library/publications/the-world-factbook/index.html and https://www.cia.gov/library/publications/cia-maps-publications/index.html NASA Earth Observatory (public domain): http://earthobservatory.nasa.gov/ Landsat: http://landsat.visibleearth.nasa.gov/ USGS EROS (Earth Resources Observatory and Science (EROS) Center) (public domain): http://eros.usgs.gov/# Natural Earth (public domain): http://www.naturalearthdata.com/ | Thank you for bringing this to our attention. Because images from Google Earth were specifically used in the data collection platform, they cannot be replaced by other non-copyrighted imagery in this figure.  Thus, we have decided to remove this figure from this manuscript and instead make reference to previous publications that include such images. |
| Editors | 6. Please upload a copy of Supporting Information SI2 [SI2 ratings.csv] which you refer to in your text on page 21. | Please see the response to the previous queries. This dataset does is too large to include as a supplemental file, so it is now publicly available at an external repository (Zenodo). |
| Reviewer 1 | This paper presents a method for optimizing volunteer contributions by determining whether a binary classification task has reached an acceptable level of certainty and further ratings are no longer necessary. Such a method allows the tasks meeting this threshold to be removed from the task pool, and enables volunteers to better use their time for other tasks. The authors presented the methodological details and used a image classification dataset to demonstrate the volunteer efforts that could have been saved by the their method. This paper is organized in a clear structure, and the authors did a great job in explaining their method. Some minor suggestions are provided as below: | Thank you for your positive view of our work. We have done our best to incorporate incorporate your suggestions which we detail below. |
| Reviewer 1 | - Introduction: The authors could add a brief discussion on a highly relevant paper: Hu, Y., Janowicz, K., & Couclelis, H. (2017): Prioritizing disaster mapping tasks for online volunteers based on information value theory. Geographical Analysis, 49, 175–198 This reference could also help the authors make the point that a better use of volunteers' time can even save lives in urgent situations such as disaster response. | Thank you for bringing this to our attention. We now make reference to it where we discuss benefits of increased efficiency at the end of the third paragraph of the discussion: "These benefits can extend beyond mere efficiency gains. For instance, in post-disaster contexts, prioritizing images that are more likely to provide useful informtion to emergency responders increases the value of volunteer work and may even help save lives [28]." |
| Reviewer 1 | - Lines 126-128: "Consider a hypothetical land cover classification task where four volunteers say they see cropland and a fifth does not detect cropland. In this case, 20% of the ratings disagree with the majority." The authors might want to revise this sentence to "... 20% of the *total* ratings disagree with the majority." for clarity; otherwise one may wonder whether the rate should be 25% (i.e., 1 no cropland and 4 cropland). | Good point! We have clarified as suggested. |
| Reviewer 1 | - One important assumption of the method presented is that the ratings are independent, i.e., one volunteer's judgement should not be affected by those of other volunteers. Such an assumption may not necessarily hold in voluntary tasks. For example, some tasks may allow one to see previous ratings; or there can be an online forum where volunteers can discuss the tasks. The authors may need to explicitly state this assumption so that users of your method are made aware of it. | We agree that this is an important point to explicitly make. Our text now clarifies in the data collection campaign section of the methods the specific design features that helped ensure that the tasks were independent in the description  We now also briefly re-iterate the relevance of this study to independent classifications in the first sentence of the discussion section. |
| Reviewer 1 | - The experiments are based on one particular dataset. Some more information about the volunteers (e.g., how these volunteers were recruited) should be provided. The authors did provide some relevant references, but having some more information about the volunteers can make this current paper self-contained. | Good idea. We have amended the description of the data collection campaign to include some basic information on recruitment, geographic origin of volunteers, and why this is unlikely to affect the results presented in this analysis:  "The volunteers were recruited mostly via appearances of project leaders in English- and German-language media [25]. Thus, many volunteers came from English- and German-speaking countries, although dozens of other countries around the world were represented. Interestingly, the classification skill of raters had very little relationship to whether the images were from their home area [25], so the international distribution of volunteers has little possibility to impact the results presented here." |
| Reviewer 2 | Dear Authors, thanks for submitting this article. Overall it is well written and covers an interesting topic. I have a few comments for things you might be able to improve. | Thank you for your positive feedback. We have addressed your specific points below. |
| Reviewer 2 | First, starting with the data availability. Maybe it's just that I didn't get the right files forwarded from the editorial manager or overlooked it. Is the data you used for this research somewhere available? Ideally you could put in on some open sharing platform, as the data might be an interesting field of study also for other scholars. | Sorry for the confusion over the data. We tried to upload this as a supplement, but didn't notice that the upload failed due to the file being >100 MB. The dataset is now available at the Zenodo repository, and the DOI for the accession is now listed in both the data availability statement and the methods section. |
| Reviewer 2 | The second major concern at the current state of the manuscript is the discussion. From my point of view you should try to improve this section, for instance by better comparing your results to the results of other studies. In your current manuscript you only cite a few references. Some references you use in the discussion (e.g. [22] about OpenStreetMap) are not directly comparable to the kind of crowdsourcing you are investigating in your study. Furthermore, it would be good if you can provide more insights how your proposed method compares to other ways of improving the crowdsourcing approach, e.g. remote sensing based approaches, object detection etc. also offer some potential to reduce the overall number of volunteered classifications needed. Whereas it is clear that this is not part of your analysis, you could add this to your discussion. | Thank you for bringing these points to our attention.  Regarding the discussion section, we did not find many other published examples of similar remote sensing image classification. We agree that our system is not directly applicable to the OSM example, but still think it is relevant as wider context, particularly given the relative lack of other examples. We have expanded this part of the discussion. However, if there are specific additional publications that we have overlooked and you think we should include, please bring those to our attention!  As you note, there are many other approaches that can be used to reduce the need for volunteer image classification. We did not dwell much on them in the original manuscript because we started from the assumption that the task at hand was something that (at least with current technology and our team's programming skill level, etc.) other approaches were not suitable for. Thus, our goal is to develop and analyze systems to apply in these particular circumstances, and avoid the enormous literature on automated approaches to these sorts of classification problems.  We now mention this more explicitly in the discussion. |
| Reviewer 2 | Regarding your results I have one main difficulty in properly understanding them. Do 59.1% refer to the overall work or does it only apply to the images for which work has been removed? It might be good to add a figure which shows the overall gain of your approach, e.g. by showing the overall number of classifications and the number of classification you identified would have been needed. The 59% can be misleading somehow as there is a different number of raters per image and this number doesn't follow a clear pattern. (Please correct me if I'm wrong, but this is what I remember from one of your previous papers) | Thank you for bringing this to our attention. The 59.1% refers to the overall work. We have now reworded the sentence where these calculations appear to begin with "This approach would have eliminated the need for 59.1% of all ratings performed by volunteers in the campaign analyzed here …"  I am receptive to the idea that a good figure could help clarify these calculations and findings. I tried a few different ways of showing distributions of un-needed ratings performed by volunteers in Cropland Capture, which resulted in Figure 4. I have no delusion that this is a good figure, but it is less bad than anything else I could think of. If you have some other suggestions, I would be receptive.  Regarding the 59% figure, you are absolutely correct that one should not necessarily expect to expect to see similar benefits in all cases. It does indeed depend on the quirks of the campaign and how it was implemented. We have made some edits to emphasize that this is a study of one particular case's potential benefits. Most notably, the first paragraph of the discussion now ends with: "The potential benefits demonstrated in this case study depend on particular details of how the Cropland Capture game was implemented, including the difficulty of the tasks and the distribution of how frequently individual images were assigned. However, we have no reason to suspect that the benefits seen here are an extreme case in either direction." |
| Reviewer 2 | In the discussion you also state that your approach would "have allowed around 2.5 times as many images to have been classified with no additional volunteer effort." (L332). Maybe it's obvious but could you highlight again how this 2.5 are derived. You mention it in the results section as well. Maybe there you can elaborate more. | Good suggestion. We now show our work for this calculation in the results section. |
| Reviewer 2 | Finally, it might be good to further highlight some limitations of your approach. In your discussion section you don't mention any, but I'm pretty sure that there will be some. In you proposed approach (but similar also for simple majority agreement) you don't consider user characteristics or the location of the tasks. Both aspects might also have an impact on the classification quality. Could these additional information be included in an approach such as you presented? | On re-reading our discussion, we see that our treatment of limitations was mostly implicit. We have made some changes to make it clearer what we view the limitations as being, and also discuss possible extensions of the logic presented here (including the complexity that comes with them).  We have actually analyzed user characteristics, especially where in the world they are from relative to the images' origins, but also professional background, in a previous study [reference 25], and found that at least for this particular task, they made remarkably little difference. This was mentioned in passing in the introduction of the first submitted text, and we now reiterate and expand on this point (including that it should not automatically be assumed true in other tasks) in the discussion. All of this is included in a new paragraph following the benefits paragraph in the discussion. |
| Reviewer 2 | Some quick comments regarding the Figures: It might be good to add a legend to Figure 1 and Figure 3 so that readers can understand easier what the colors refer to. | Good suggestion! We have implemented it. |
|  | In Figure 4 there seems to be a pattern (like a seasonality) in the data. how can this be explained? Is this a limitation of the proposed method? | I think you are referring to the jagged pattern in panels A-C? This is a result of allowing responses of 'maybe'. Thus, you can get an outcome of 9 yes to 0 no after 11 votes if there were two ratings of 'maybe'. This is what is going on in the lower areas between the peaks. We actually attempted (and clearly failed!) to explain this in the last two sentences of the figure legend; your comment has convinced us that this is just confusing to include and doesn't have any real relevance to the analysis as it is carried out here. Thus, we have re-run the analyses without the 'maybe' ratings, so the numbers presented in our revised manuscripts are a bit different than the first submitted version. This also turns the jagged pattern from the previous Figure 4 (now Figure 3) into a comb-like pattern for classification at cropland or non-cropland. This is due to maybe classifications being eliminated while it remains true that only certain combinations of numbers can get an image over the classification threshold. |
| Reviewer 2 | Another quick one about "disagreement rate" (L125). Is this your own definition or a commonly agreed one? I think it makes sense, but are not sure where the definition comes from. | Good question. I find this terminology in some of our previous papers, and I can't remember this term having another source, but also can't say for certain that it doesn't. I have tweaked the wording where this term first appears so that it is clear that this is what we mean in this particular study, without giving the appearance that we claim no one has used this term before. |
| Reviewer 2 | Finally, a more personal question. You show interesting results. Still the number if ratings needed for such a "simple" task seems rather high to me. For instance, when mapping settlements with MapSwipe one usually uses much less number or ratings per image. Usually between 3-5. Is this a "problem" or is the task of identifying buildings just a bit easier than identifying cropland? | I think there are at least two facets to the answer. The first part, as you suggest, is that identifying cropland is sometimes tricky. As you can see in our results, many places in the world are very clearly identifiable as cropland or non-cropland, but there are many other places that are extremely difficult to evaluate in this regard (in addition to the figures here, see our previous papers referenced in this manuscript). The second part is that we are applying a more stringent standard here than might be applied in some other circumstances. For instance, a binomial test to determine if a binomial result distribution differs from an expected outcome of .5 gives a p-value of .0625 with only 5 consistent outcomes. We are asking whether at least 95% of the distribution of the underlying parameter (the .5 value in this example) is between .8 and 1.0 (hence the Bayesian approach), which is stricter evidence about the certainty of the reviews. This level of certainty might not be appropriate for all applications, which is why we encourage readers to modify and use our code as suited to their needs. |
